# Investigating the impact of chilling temperature on male *Aedes aegypti* and *Aedes albopictus* survival

**DOI:** 10.1371/journal.pone.0221822

**Published:** 2019-08-27

**Authors:** Nicole J. Culbert, Jeremie R. L. Gilles, Jérémy Bouyer

**Affiliations:** 1 Insect Pest Control Laboratory, Joint Food and Agriculture Organization of the United Nations/International Atomic Energy Agency Programme of Nuclear Techniques in Food and Agriculture, Vienna, Austria; 2 Institute of Integrative Biology & the Centre for Genomic Research, University of Liverpool, Liverpool, Merseyside, England, United Kingdom; 3 CIRAD, UMR ASTRE CIRAD-INRA « Animals, Health, Territories, Risks and Ecosystems », Campus international de Baillarguet, Montpellier, France; Swedish University of Agricultural Sciences, SWEDEN

## Abstract

In genetic control programmes, including the sterile insect technique (SIT), it is crucial to release insects of the highest quality with maximum survival. It is likely that male mosquitoes will follow the trend of other insects in SIT programmes and be stored, transported and eventually released under chilled conditions. The aim of our study was to investigate the impact of different chilling temperatures on male *Aedes aegypti* and *Ae*. *albopictus* survival by exposing them to a range of temperatures for different durations. *Ae*. *aegypti* were found to be less sensitive to the impact of chilling, with only 6°C causing a marginal decrease in survival in comparison to non-chilled controls. Conversely, *Ae*. *albopictus* displayed a significantly reduced survival at all chilling temperatures even when exposed for a short time. In both species, longer exposure to low temperatures reduced survival. Our results uncovered that *Ae*. *albopictus* are more sensitive to chilling, regardless of the temperature, when compared to *Ae*. *aegypti*. Such results indicate differences in thermal tolerances between species and the necessity of conducting experiments on a species by species basis when determining temperature limits for any insect destined for release as part of a genetic control programme.

## Introduction

The global burden of vector-borne diseases is increasing with mosquito borne diseases responsible for more than 700,000 deaths each year [1]. With traditional vector control methods such as insecticides becoming less effective due to a build up of resistance in wild populations, alternative vector control tools are needed urgently [2]. Genetic control techniques such as the sterile insect technique (SIT) has seen reignited interest in recent years as a potential method to control mosquitoes as part of an area-wide integrated pest management (AW-IPM) strategy, having been used successfully against various plant and animal pests for over six decades [3].

Significant progress has been made in the last decade towards taking mosquito SIT to the operational level [4]. Mass rearing methodology has been developed and standardized, including larval rearing equipment [5, 6], larval diet [7] and more recently a rapid quality control device based on flight ability [8]. However, distinct gaps still remain in the literature, especially regarding the post-pupal irradiation stages or the handling, transport and release of sterile male mosquitoes. All of which are essential components of A SIT programme and, if not addressed correctly, could impact sterile male quality and survival [9]. Historically, the aerial release of sterile insects was carried out by deploying biodegradable release cartons filled with known quantities of insects which minimised the impact of handling and thus preserved quality [10]. Today, sterile insects such as fruit flies (*Ceratitis capitate*) and tsetse flies (*Glossina palpalis gambiensis*) are released via a new continuous release system, releasing the chilled adults at a defined rate per surface area [11]. During the transportation phase, prior to an aerial release, fruit flies and tsetse flies remain immobilised within a temperature range of 8 to 10°C [9, 12],

To ensure the success of any release campaign, it is essential to release insects with a maximal quality. As mosquitoes are small bodied poikilotherms, it comes as no surprise that temperature is consistently noted as a key element impacting survival [13]. The phenomenon of inducing immobilisation at a species-specific temperature is referred to as the critical thermal minimum (CT_min_) or knockdown temperature where the righting response is lost and the insect is unable to stand up or cling to a surface. This precedes a stage known as a chill-coma where a reversible cessation of movement occurs [14]. It has been shown that exposing insects to temperatures out of their normal range can cause stress and in turn reduces their quality and competitiveness [15]. Thus, it is fundamental to assess the effects of immobilizing male mosquitoes across an array of temperatures to define a suitable range within which any potential negative impact is mitigated. It is documented within the literature that activity will cease in *Aedes aegypti* at temperatures below 10°C [16, 17].

Previous studies conducted within our laboratory have already suggested a suitable temperature range in which to transport *Anopheles arabiensis* [18]. We endeavoured to build on this research and define an optimal temperature range for storing and transporting immobile male *Ae*. *aegypti* and *Ae*. *albopictus*. We aimed to determine the temperature thresholds above which, immobilisation would not occur and below which reversible damage arises and thus impedes subsequent survival and quality. We exposed male *Ae*. *aegypti* and *Ae*. *albopictus* to a range of cold storage temperatures for various time periods and monitored their survival post-chilling.

## Materials and methods

### Mosquito colony sources and mass rearing procedures

The strains of *Ae*. *aegypti* and *Ae*. *albopictus* used in the present study were sourced from Juazeiro, Brazil and provided by Biofabrica Moscamed and Rimini, Italy and provided by the centro agricoltura ambiente (CAA) in Crevalcore, Italy respectively. Both *Ae*. *albopictus* and *Ae*. *aegypti* have been subsequently reared in the Food and Agricultural Organisation/ International Atomic Energy Agency (FAO/IAEA) Insect Pest Control Laboratory (IPCL) in Seibersdorf, Austria since 2010 and 2012 respectively without further colony regeneration.

Adults were maintained under controlled temperature, relative humidity and light regimes (27 ± 1°C, 70 ± 10% RH, 12:12 h light:dark (L:D) photoperiod, with two one-hour twilight periods simulating dawn and dusk as described in [19]. Eggs used for all experiments were generated and hatched based upon standardised guidelines developed at the IPCL [20]. Larvae were mass-reared in a climate controlled room with temperature and RH held constant at 30 ± 1°C, 70 ± 10% RH, respectively. Larvae were reared in mass rearing trays with approximately 18,000 first instar (L_1_) per tray in 5 l of deionized water. 7.5% IAEA diet was administered daily (50 ml on day 1, 100 ml on day 2, 150 ml on day 3, 200 ml on day 4 and 50 ml from day 5 onwards) [21]. Pupae were sexed mechanically using a Fay-Morlan [22] glass plate separator subsequently redesigned by Focks (John W. Hock Co., Gainesville, FL, USA [23]), prior to further examination under a stereomicroscope for further accuracy. Adults were maintained in small plastic Bugdorm cages (Bugdorm, Taipei, Taiwan; 15 x 15 x 15 cm) in batches of 30 males per cage with continuous access to a 10% sucrose solution. The experiment commenced on day 2 post-emergence.

### Determining the experimental temperature range

Short preliminary experiments were conducted to determine experimental temperatures. Bugdorms of 30 adult *Ae*. *aegypti* and *Ae*. *albopictus* were placed inside a climate chamber (Sanyo MLR-351H, Osaka, Japan) with a Hero4 GoPro camera for 20 min to test each temperature across the range of 2–14°C. As immobilisation did not occur above 12°C, a narrower range of 2–10°C was selected for further investigation. To monitor the temperature inside the climate chambers, Data loggers (Onset Hobo data loggers, Bourne, MA, USA) were placed inside.

### The impact of chilling temperature on survival

A Bugdorm containing 30 adult male *Ae*. *aegypti* (3 replicates for each temperature and duration) were maintained inside a climate chamber programmed to 4, 6, 8 or 10°C, RH 50%, for 1, 4, 8 or 24 h; control adults were maintained at 25 ± 1°C. Immediately after cold exposure, experimental males were returned to 25 ± 1°C. The experiment was repeated for *Ae*. *albopictus* but at 2, 4, 6 and 10°C. A slightly lower temperature (2°C) was selected for *Ae*. *albopictus* as we expected that they would be more tolerant to cold temperatures. Immediate mortality was assessed following immobilisation and survival monitored for a further 14 days, with dead adults removed daily. Survival was not monitored beyond 14 days as survival post-release is not likely to exceed this timeframe.

### Statistical analysis

Binomial linear mixed effect models were used to analyze the impact of the various temperatures and durations on survival rates on day 15 post-exposure (response variables). Temperature and duration were then used as fixed effects and the repetitions as random effects. The temperature of 25°C and the duration of one hour were set as reference levels (control) in all models and other treatments were compared to these values. The significance of fixed effects was tested using the likelihood ratio test [24, 25] and are reported in Tables 1 and 2.

**Table 1:** Fixed-effects coefficients of a mixed-effect binomial model of the impact of temperature and duration of the survival of male *Aedes aegypti* on day 15 post-exposure.

Fixed effects	Value	Std. Error	z-value	p-value
Intercept	3.55420	0.28728	12.372	2e-16
4°C	-0.52051	0.32859	-1.584	0.1132
6°C	-0.64037	0.32253	-1.986	0.0471
8°C	-0.18227	0.3494	-0.522	0.6019
10°C	-0.33936	0.33901	-1.001	0.3168
Duration	-0.04433	0.01007	-4.403	1.07e-05

**Table 2:** Fixed-effects coefficients of a mixed-effect binomial model of the impact of temperature and duration of the survival of male *Aedes albopictus* on day 15 post-exposure.

Fixed effects	Value	Std. Error	z-value	p-value
Intercept	2.924461	0.237463	12.315	2e-16
2°C	-0.838245	0.270320	-3.101	0.00193
4°C	-0.625690	0.278129	-2.250	0.02447
6°C	-0.625690	0.278129	-2.250	0.02447
10°C	-0.625690	0.278129	-2.250	0.02447
Duration	-0.019357	0.008331	-2.323	0.02015

## Results

### The effect of temperature and duration on male *Aedes* survival

**Fig 1:**
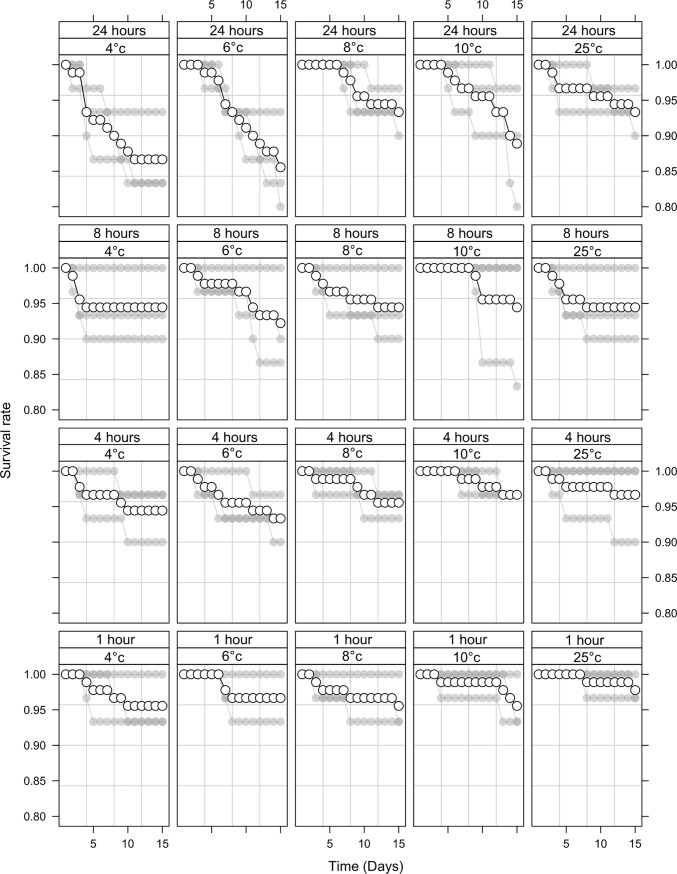
Mean (± standard error, SE) % survival of adult male *Aedes aegypti* 15 days following immobilisation at 4, 6, 8 and 10°C for 1, 4, 8 or 24 h. Mean values are represented by white circles with a black outline. Each series of grey dots represents a repetition (one cage. of 30 adult males).

In *Ae*. *aegypti*, temperature did not significantly reduce survival 15 days after immobilisation when exposed to all temperatures for one hour (Fig 1; Table 1, p > 0.05) except at 6°C where a marginally significant effect was observed (p = 0.05). With an increase in duration of chilling however, there was a subsequent decrease in survival at all temperatures (p < 10^−3^).

**Fig 2:**
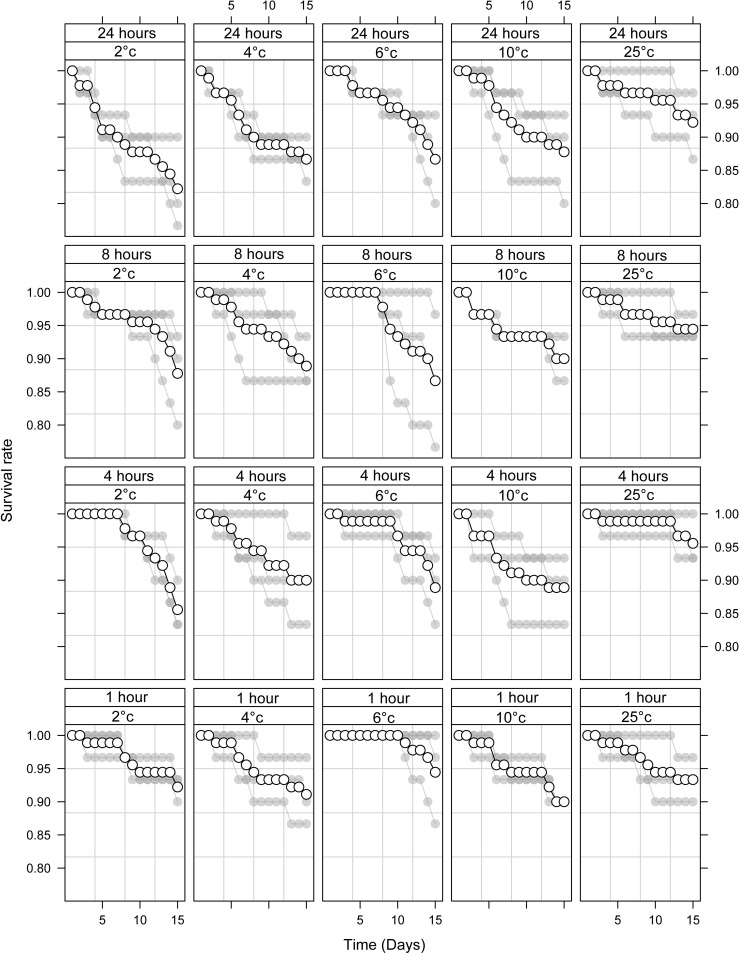
Mean (± standard error, SE) % survival of adult male *Aedes albopictus* 15 days following immobilisation at 2, 6, 8 and 10°C for 1, 4, 8 or 24 h. Mean values are represented by white circles with a black outline. Each series of grey dots represents a repetition (one cage. of 30 adult males).

In *Ae*. *albopictus*, there was a significant decrease in survival 15 days after immobilisation at all temperatures to which they were exposed (2, 4, 6 and 10°C, Fig 2; Table 2, p < 0.03), with duration again further reducing survival with each increase in duration (p = 0.02). We therefore suggest that *Ae*.*aegypti could be stored immobile between 7 and 10*°C without negatively impacting their survival, at least for short durations, yet further testing is warranted for extended periods of immobilisation. We would be keen to repeat our experiments with *Ae*. *albopictus*, especially at the higher end of the immobilisation threshold (6–10°C) to see if our results hold true and if there are differences between strains.

## Discussion

The results of our study show stark differences in survival post-chilling between *Ae*. *aegypti* and *Ae*. albopictus. Our previous study highlighted that male *An*. *arabiensis* could be held at 2°C for up to 8 hours or between 4 and 10°C for up to 24 hours without significantly impacting subsequent survival 14 days after immobilisation, except a marginally significant reduction at 6°C which might be due to experimental uncertainties. This result is consistent with what was found in an earlier study where male *Ae*. *aegypti* were exposed to 0, 4, 8 and 10°C for a period of 2 hours with post-chilling survival monitored for a period of 15 days [8]. Only exposure to 0°C was found to significantly decrease survival whilst 4, 8 and 10°C did not as per our current study. The susceptibility of *Ae*. *albopictus* to all chilling temperatures is in stark contrast.

It is postulated that the expanding range of *Ae*. *albopictus* is attributed to its ability to survive at low temperatures whilst the more limited spread of *Ae*. *aegypti* is due to its inability to withstand colder conditions. This has been shown in larval survivorship in the 2 species exposed to low temperatures with *Ae*. *albopictus* displaying a higher survival that *Ae*. *aegypti* [26]. However, a review of the literature by Brady et al, also concluded that *Ae*. *aegypti* have a greater tolerance to lower temperatures when compared to *Ae*. *albopictus*, again in direct contrast to their observed geographic distribution [27]. This result may be explained when considering the egg stages of each species. The eggs of *Ae*. *albopictus* eggs are capable of undergoing diapause and thus allowing the species to overwinter [28]. On the other hand, *Ae*. *aegypti* eggs show far less adaptation to survive beyond their normal thermal limits [29]. Thus, there may be a stronger selection pressure in adults to withstand a wider temperature range to resist diurnal and inter-seasonal variations in temperature [27]. A further, more recent review by Schmidt et al, investigating the relationship between humidity and adult survival with temperature as a modifying effect, concluded that the lowest mortality risk for *Ae*. *albopictus* and *Ae*. *aegypti* was at 21.5 and 27.5°C respectively [30]. Despite the optimum temperature being lower in *Ae*. *albopictus*, it was noted that *Ae*. *aegypti* had a survival advantage under most of the tested conditions. Therefore, accounting for humidity may offer an explanation for the differing results reported in the reviews by Schmidt and Brady. Humidity also remained constant during our studies and thus, it may be that *Ae*. *albopictus* may survive better when chilled at lower temperatures if the level of humidity is adjusted. Further investigation is necessary. It is also worth noting that both reviews were based upon adult female survival whilst our studies only involved males.

Insects are well known to display a high level of variability when it comes to cold tolerance, both between and within species. This phenomenon has been reported in *Aphidiinae* [31] and *Trichogramma* species [32] with even inter-population variability in cold tolerance reported in the latter [33]. Cold tolerance can vary within one population due to epigenetic changes. This is especially true of *Ae*. *albopictus*, where a local short-term mechanism of the heritable trait of cold hardiness has been suggested for its successful spread into cooler climates [34]. It may be that the strain of *Ae*. *albopictus* we used for our study, which originated from Rimini, Italy, was less cold tolerance than other strains. The results from our studies concerning both *Ae*. *aegypti* and *Ae*. *albopictus* highlighted how determining conditions for one species does not mean it can be inferred for another. *Aedes* species appeared to be less tolerant to low temperatures, even for short durations than *An*. *arabiensis*, with *Ae*. *albopictus* displaying a greater sensitivity than *Ae*. *aegypti*. We would advise to maintain an immobilisation temperature of between 7 and 10°C when storage and transporting *Ae*. *aegypti* for short periods of time. With the recent development of our novel quality control tool [8], it may be of value to repeat these studies and follow up with flight ability tests to ascertain if chilling temperature is having a similar effect on their quality, especially in *Ae*. *albopictus*. Such a high natural variability between species therefore means that individual studies are necessary to determine species-specific parameters for storage and transportation for any insect and, most likely, any strain when considering them for release as part of a genetic control programme.

## Conclusions

The results of our study highlight a variation in thermal tolerance between species and how determining limits for one cannot be inferred for another. It is therefore necessary to conduct such studies on a species by species basis in order to ensure that the quality of the insect destined for release is of the highest quality.

## Supporting information

S1 Appendix Experimental data. All data used to generate the results for this study.
(XLSX)

## Abbreviations

SIT: Sterile insect technique
RH: Relative humidity
IPCL: Insect Pest Control Laboratory
FAO: Food and Agricultural Organization
IAEA: International Atomic Energy Agency
LD: light:dark
